# A Novel Prognostic Chemokine-Related lncRNAs Signature Associated with Immune Landscape in Colon Adenocarcinoma

**DOI:** 10.1155/2022/2823042

**Published:** 2022-11-03

**Authors:** Xinglong Dai, Menghua Zeng, Zhen Huang, Jun Zhang, Zhengqiang Wei, Ziwei Wang

**Affiliations:** Gastrointestinal Surgical Unit, The First Affiliated Hospital of Chongqing Medical University, Chongqing, China

## Abstract

Chemokines have been reported to be involved in tumorigenesis and progression and can also modulate the tumor microenvironment. However, it is still unclear whether chemokine-related long noncoding RNAs (lncRNAs) can affect the prognosis of colon adenocarcinoma (COAD). We summarized chemokine-related genes and downloaded RNA-seq and clinical data from The Cancer Genome Atlas (TCGA) database. A total of 52 prognostic chemokine-related lncRNAs were screened by univariate Cox regression analysis; patients were grouped according to cluster analysis results. Lasso regression analysis was applied to determine chemokine-related lncRNAs to construct a risk model for further research. This study first investigated the differences between the prognosis and immune status of two chemokine-related lncRNAs clusters by consensus clustering. Then, using various algorithms, we obtained ten chemokine-related lncRNAs to construct a new prognostic chemokine-related lncRNAs risk model. The risk model's predictive efficiency, validity, and accuracy were further validated and determined in the test and training cohorts. Furthermore, this risk model played a vital role in predicting immune cell infiltration, immune checkpoint gene expression, tumor mutational burden (TMB), immunotherapy score, and drug sensitivity in COAD patients. These findings elucidated the critical role of novel prognostic chemokine-related lncRNAs in prognosis, immune landscape, and drug therapy, thereby providing valuable insights for prognosis assessment and personalized treatment strategies for COAD patients.

## 1. Introduction

Colon adenocarcinoma (COAD) is one of the digestive system's most common and deadly cancers [1]. Colonoscopy is an early screening method that can effectively prevent COAD's occurrence. Still, its insidious onset, high malignancy, and easy metastasis often lead to a worse prognosis [2]. COAD is characterized by high biological invasiveness and specific radio- and chemo-resistance, resulting in high recurrence rates and tumor progression [3]. With the advancement of surgery, chemotherapy, targeted therapy, and novel immunotherapy, the efficacy and survival of COAD patients have improved significantly. However, advanced COAD patients are still prone to recurrence and metastasis, and only a small number of patients benefit from the above treatment. The common factors are epigenetic changes and accumulation [4]. Thus, identifying effective prognostic biomarkers and their underlying functional characteristics may contribute to accurate survival prediction and optimal treatment of COAD patients.

Long noncoding RNAs (lncRNAs) are composed of sequences >200 bp and lack protein-coding capacity [5]. Many lncRNAs are involved in gene regulation and various biological functions at the transcriptional, posttranscriptional, and epigenetic levels, including the recent discovery that some lncRNAs can encode small peptides/proteins [6]. Accumulating evidence showed that aberrant expression of lncRNAs is not only associated with tumor malignancy but many chemically modified lncRNAs have been validated in various cancers [7, 8]. There may be interactions between these modifications, with some competitive compensation. Notably, multiple lncRNAs have been identified as prognostic biomarkers that can be used for tumor subtype identification, treatment response prediction, and modulation of immune status [9]. Studies on the clinical and biological functions of tumor-related lncRNAs are still being reported.

Chemokines are a class of cytokines with the chemotactic activity that has been reported to affect tumorigenesis and serve as potential therapeutic targets. The dysregulation of chemokines and chemokine receptors has been closely associated with tumor progression, including COAD [10]. For example, EMT-mediated CXCL1/5 can modulate resistance to anti-EGFR therapy in colorectal cancer, and CXCL1/5 may be a potential serum biomarker for predicting colorectal cancer resistance to EGFR therapy [11]. Another chemokine, CCL11, exacerbates colitis and inflammation-related colon tumorigenesis [12]. Chemokines can also affect the infiltration of various immune cells and the tumor microenvironment, thereby affecting tumor progression. CXCL14 may act as an important factor in determining the immune microenvironment in gliomas, thereby promoting antitumor CD8+ T cell responses [13]. CCL24 can promote multiple cancer progression, including COAD, through M2 macrophage polarization, angiogenesis, invasion and migration, and eosinophil recruitment [14]. lncRNAs are also involved in the chemokine regulation of colon tumors; for example, chemokine ligand 5 is engaged in tumor-associated dendritic cell-mediated colon cancer progression through noncoding RNA MALAT-1 [15]. These reports suggested that chemokine-related genes or lncRNAs play critical roles in cancers, especially tumor microenvironments (TMEs). Based on this, studying the characteristics of chemokine-related lncRNAs is of great significance for understanding how lncRNAs affect the prognosis, immune status, and tumor-related treatment of COAD patients.

The tumor microenvironment (TME), as an essential component of malignancies, plays multiple roles in tumorigenesis, progression, metastasis, recurrence, and therapy resistance [16]. Complex interactions between tumor cells and the TME can promote tumor progression. Xiao et al. found that the tumor-infiltrating immune cells (TIICs) in the TME environment are highly valued in predicting cancer prognosis [17]. Recent studies have reported that immune checkpoint proteins are associated with TME and can regulate immune signaling pathways to evade immune responses and promote tumor progression [18]. Furthermore, some articles showed that tumor mutational burden (TMB) was markedly correlated with 21 tumor patients, and there were specific differences in TMB among different tumors [19]. Jiang et al. reported that immune cell infiltration and TMB scores could synergistically predict survival in gastric cancer patients [20]. To elucidate how the chemokine-related lncRNAs network affects the TME and TMB, it is necessary to understand the crosstalk between different lncRNA patterns. Understanding this network may provide essential insights into COAD patients' survival, tumor immunity, and new therapeutic options.

This study first investigated the differences between the survival outcomes and immune status of two chemokine-related lncRNA clusters by consensus clustering. We then constructed a new risk model of prognostic chemokine-related lncRNAs that played a crucial role in predicting immune cell infiltration, immune checkpoint gene expression, TMB, immunotherapy score, and drug sensitivity in COAD patients. Furthermore, we analyzed the prognostic value and expression level of each lncRNA in this model in COAD patients. This study will help to explore the role of prognostic chemokine-related lncRNAs and provide new clues for the occurrence, progression, and treatment of COAD.

## 2. Materials and Methods

### 2.1. Data Acquisition and Processing

Transcriptome sequencing and clinical data of COAD patients were downloaded from The Cancer Genome Atlas (TCGA-COAD) database. Raw data were collected from 473 tumor samples and 41 normal tissues using Perl software (version 5.32.1). We extracted expression data for lncRNAs and mRNAs by annotating gene symbols using human GTF files. We excluded COAD patients with no overall survival value or missing status to reduce statistical bias. The relevant clinical information involved age, grade, stage, TNM stage, survival status, and survival time, as shown in Supplementary Table S1.

### 2.2. Obtaining the Prognostic Chemokine-Related lncRNAs

Based on previous studies on chemokines, we obtained 64 chemokine-related molecules (Supplementary Table S2) [21, 22]. The chemokine-related lncRNAs were screened and extracted using Pearson correlation analysis with the criteria of |Pearson R| > 0.3 and *p* < 0.001. lncRNAs associated with one or more of the 64 chemokines regulators were defined as chemokine-related lncRNAs. After obtaining chemokine-related lncRNAs, we combined the survival status and survival time of COAD patients with lncRNA expression data. Univariate Cox regression analysis was performed to determine the prognostic chemokine-related lncRNAs with a *p* value of 0.05 via the survival package (Supplementary Table S3). In addition, differences in the expression of prognostic chemokine-related lncRNAs between tumor and normal samples were tested using Wilcoxon signed rank and shown as boxplots.

### 2.3. Analysis of COAD Subtypes Defined by Prognostic Chemokine-Related lncRNAs

The consensus clusters were determined based on the expression and underlying biological features of prognostic chemokine-related lncRNAs by the ConsensusClusterPlus package (pfeature = 1, resample rate = 0.8, and iterations = 50). The optimal *k* value (*k* = 2) was determined to obtain relatively stable clusters, namely, clusters 1 and 2. The prognostic value of COAD patients in subgroups was analyzed using the Kaplan–Meier method and log-rank tests. The Chi-square test or Fisher's exact test was utilized to determine the relationship between clinical characteristics and clusters. In addition, the differential expression and clinical features of prognostic chemokine-related lncRNAs were displayed using the pheatmap package.

### 2.4. Immune Infiltration Level Analysis among the Subgroups

We used the CIBERSORT algorithm to assess immune cell infiltration, converting a matrix of gene expression in the sample into the content of immune cells, with a *p*value < 0.05 indicating reliable cellular composition (Supplementary Table S4). Immune, stromal, and ESTIMATE scores were calculated to compare immune infiltration between the subgroups using the ESTIMATE algorithm by the limma and ggpubr packages (Supplementary Table S5). Differences in immune cell infiltration between the two clusters were verified using the vioplot package. Differences in immune checkpoint inhibitor molecules between subgroups were assessed using the Wilcoxon test. In addition, the coexpression correlation between chemokine-related lncRNAs and immune checkpoint inhibitors was detected by corrplot and limma packages.

### 2.5. Construction and Validation of Risk Model

The 52 prognostic chemokine-related lncRNAs were used to construct the risk model by LASSO regression analysis. COAD patients with survival data were randomly divided into training and testing groups using R caret, glmnet, surviner, and the survival packages. The training cohort was used to build the risk model, and the entire cohort and the test cohort were used to validate the risk model (Supplementary Table S6). We identified ten chemokine-related lncRNAs to build a risk model. The risk score formula was as follows: risk score = ∑_*i*=1_^*n*^Coefi∗Expi, where Coefi represents the coefficient, and Expi represents the expression value of chemokine-related lncRNA. The training and test groups were divided into the high-risk and low-risk groups based on the median score. The prognostic significance of the high- and low-risk groups was assessed using the survival package. Receiver operating characteristic (ROC) curves were used to evaluate the predictive accuracy and validity of the model via the “survivalROC” package. We plotted risk curves for COAD patients in the training and test groups and evaluated survival status and risk with the training and testing groups.

### 2.6. Independent Prognostic Value of the Risk Model and Pathways

To investigate whether risk score might be an independent prognostic factor, and the clinical characteristics of COAD patients by univariate and multivariate Cox regression analysis, ROC curve was used to verify the clinical application value of the risk model. The predictive power of risk scores in age, sex, grade, stage, and TNM stage subgroups was validated by stratified survival analysis. We constructed a nomogram to predict the survival time of COAD patients using the “survival” and “regplot” R packages, and the accuracy of the nomogram was assessed by obtaining a calibration curve using the “rms” package. The hallmark (h.all.v6.2.entrez.gmt) and KEGG were acquired from the Molecular Signatures Database (MSigDB) using GSEA V3.0 and the GSEABase, and reshape2 packages. The false discovery rate FDR < 0.05 and *p* < 0.05 was statistically significant. Furthermore, the potential biological mechanisms of high- and low-risk groups were investigated using gene set variation analysis (GSVA).

### 2.7. Correlation of the Risk Model with the TME and Immune Cell Infiltration

The immune cell infiltration in all tumor samples was calculated using different software (XCELL, TIMER, QUANTISEQ, MCPCOUNTER, EPIC, CIBERSORT-ABS, CIBERSORT); a *p*value < 0.05 indicated that the inferred cellular composition is reliable (Supplementary Table S7). The immune cell correlation analysis showed which immune cells were associated with the patient's risk score and obtained a correlation bubble plot using the scales and tidyverse packages. We examined the differences between the two groups for immune, stroma, and ESTIMATE scores by Wilcoxon's test and plotted the results as vioplot. The correlation analysis of prognostic chemokine-related lncRNAs and immune checkpoint inhibitory molecules were detected using the R packages “limma,” “reshape2,” “ggplot2,” and “ggpubr,” and then plotted by the corrplot package. Furthermore, single-sample gene set enrichment analysis (ssGSEA) was utilized to evaluate the differences in immune-related pathways between high-risk and low-risk groups by using the R packages “limma,” “GSVA,” “GSEABase,” “ggpubr,” and “reshape2”.

### 2.8. Correlation between the Risk Model and Tumor Mutation Burden

Tumor mutation burden (TMB) data of COAD was downloaded from the TCGA database. The COAD patients were classified into high or low TMB groups based on median values. The correlation between the TMB and risk model was verified by using “ggpubr,” “reshape2,” and “ggplot2” packages. We also visualized the top 20 genes with the highest mutation frequency in high-risk (Supplementary Table S8) and low-risk groups (Supplementary Table S9) using the maftools package. Survival differences among patients with different TMB statuses and risk scores were examined by survival analysis.

### 2.9. Clinical Value of the Risk Model in Immunotherapy and Targeted Drug Screening

We downloaded the data of the immunotherapy score from the TCIA database (Supplementary Table S10) and analyzed the effects of immunotherapy in high- or low-risk groups. Then, the drug sensitivities were assessed in patients with different risk groups using the limma, ggpubr, and pRRophetic packages, which predict 50% inhibitory concentration (IC50) of common drugs for COAD. Subsequently, we determined drug sensitivities in different risk groups and screened for potential therapeutic agents that might affect patient survival. Differences between groups were assessed using the Wilcoxon signed-rank test, with *p* < 0.001 as the screening criterion.

### 2.10. Tissue Sample Collection, RNA Extraction, and Quantitative Real-Time PCR

We collected 20 human COAD tissues and adjacent normal tissues from the First Affiliated Hospital of Chongqing Medical University. This study was approved by the Ethics Committee of the First Affiliated Hospital of Chongqing Medical University, and all patients signed the informed consent. Total RNA from COAD samples was extracted using the Trizol reagent (Takara, Japan) according to the manufacturer's protocol. Total RNA was reverse-transcribed to cDNA using the PrimeScript™ RT Reagent Kit (#RR037A, Takara, Japan). All primers were designed and synthesized by Sangon Biotech (Sangon Biotech, China, Supplementary Table S11). The qRT-PCR assays were performed using TB Green Premix Ex Taq II (Takara, #RR820A). The relative expression (fold change) of the target molecules was calculated using the 2 − ΔΔCT method. GAPDH was the internal control.

### 2.11. Statistical Analysis

All data were analyzed, and images were generated using R (version 4.1.3) and GraphPad Prism (version 8.03, GraphPad Software Inc., USA). Pearson correlation test was used for the correlation analysis. Survival analyses were performed using the Kaplan–Meier method with a log-rank test. Wilcoxon signed-rank test and the Kruskal-Wallis test were used for comparison between groups. The results of PCR experiments were expressed as mean ± SD, and statistical significance was determined by paired *t*-test. A *p*value < 0.05 indicated statistical significance.

## 3. Results

### 3.1. Consensus Clustering of Prognostic Chemokine-Related lncRNAs

**Figure 1:**
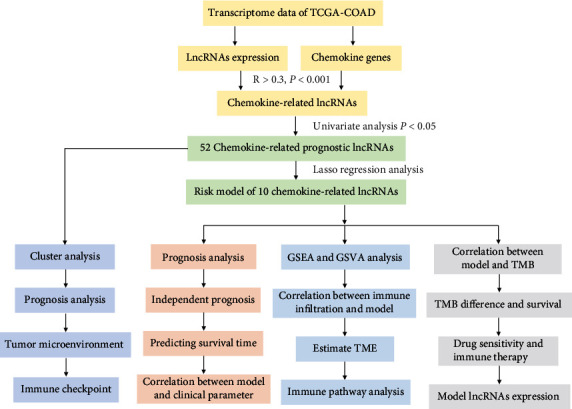
Flow chart of this study.

**Figure 2:**
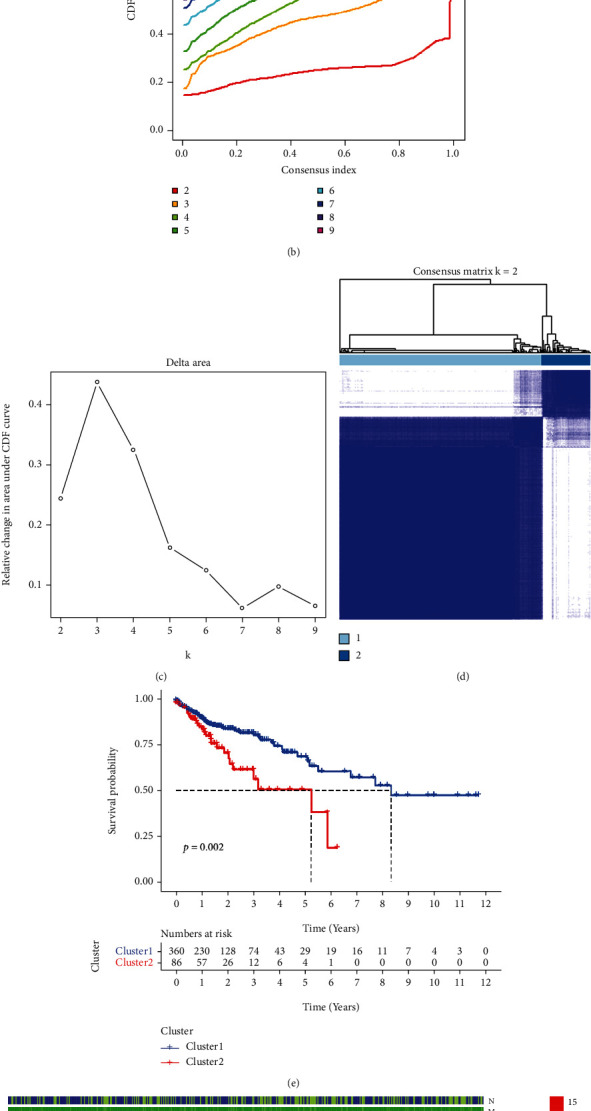
Consensus cluster analysis of chemokine-related lncRNAs in COAD. (a) Forest plot of the prognostic value of 52 chemokine-related lncRNAs in COAD. (b) Consensus clustering cumulative distribution function (CDF) for *k* = 2 − 9. (c) Relative change in area under the CDF curve for *k* = 2 − 9. (d) Consensus clustering matrix for *k* = 2. (e) Kaplan–Meier survival analysis of overall survival for COAD patients in clusters 1 and 2. (f) The heatmap of clinicopathological characteristics and lncRNAs expression in clusters 1 and 2. ^∗^*p* < 0.05, ^∗∗^*p* < 0.01, ^∗∗∗^*p* < 0.001.

This study's workflow is shown in Figure 1. After obtaining chemokine-related lncRNAs, we initially identified 52 prognostic chemokine-related lncRNAs by using univariate Cox regression analysis (Figure 2(a)). The expression of 52 prognostic chemokine-related lncRNAs in tumor and normal tissues was detected and displayed as heatmaps and boxplots (Supplementary Figures S1A, S1B). Based on the similarity in the expression of prognostic chemokine-related lncRNAs, consensus clustering showed that COAD patients were divided into 2 subgroups, the cluster stability was the best, and the CDF value was the lowest. Therefore, the lncRNAs were divided into clusters 1 and 2 (Figures 2(b)–2(d)). To assess the survival of chemokine-related lncRNAs in different clusters, survival analysis showed that patients in cluster 2 had lower overall survival than those in cluster 1 (Figure 2(e)). The heatmap showed that the clinical parameters of COAD patients in the two clusters were not significantly different (Figure 2(f)). Afterward, we found significant differences in the proportion of infiltrating immune cells (TIICs) in each COAD sample, providing clues for further investigation of chemokine-related prognostic lncRNAs in the tumor microenvironment (Supplementary Figure S1C).

### 3.2. Analysis of the TME and Immune Checkpoint Molecules Clusters 1 and 2

**Figure 3:**
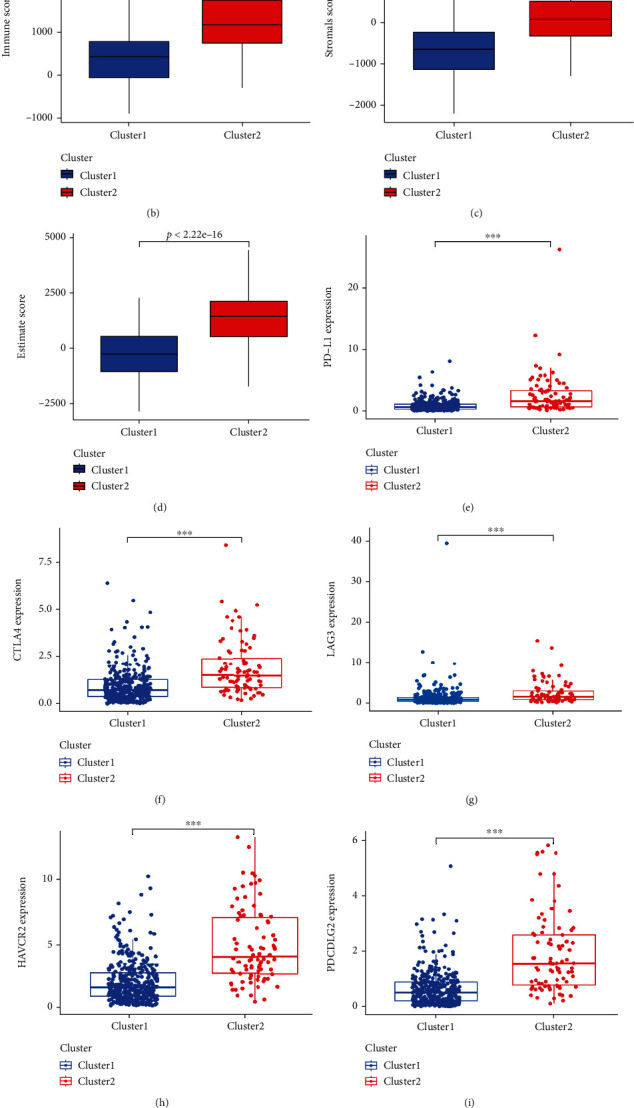
Analysis of the immune cell infiltration and TME in different clusters. (a) Infiltration levels of 22 immune cell types in clusters 1 and 2 subtypes. (b–d) Immune (b), stromal (c), and ESTIMATE (d) scores among subgroups in COAD. (e–k) Boxplot of the expression levels of PD-L1 (e), CTLA4 (f), LAG3 (g), HAVCR2 (h), PDCD1LG2 (i), SIGEC15 (j), and TIGIT (k) in cluster 1 and cluster 2 in COAD. (l) Correlation of chemokine-related lncRNAs with differentially expressed immune checkpoint molecules. ^∗^*p* < 0.05, ^∗∗^*p* < 0.01, ^∗∗∗^*p* < 0.001.

We initially analyzed the differences in immuneScore and immune cell infiltration and exhibited a vioplot between clusters 1 and 2. Immune cells such as Neutrophils and T cells follicular helper were highly clustered in cluster 2, whereas Mast cells resting, Dendritic cells resting, and T cells CD4 memory resting were highly aggregated in cluster 1 (Figure 3(a)). Based on the ESTIMATE algorithm, the immuneScore, stromalScore, and ESTIMATEScore in cluster 2 were dramatically higher than in cluster 1 (Figures 3(b)–3(d)). Then, we verified the correlation among 22 immune cells in COAD. For example, immune cells such as Macrophages M0, Mast cells activated, NK cells resting, and T cells CD4 memory activated were markedly negatively correlated with cells such as T cell CD8, NK cells activated, Mast cells resting, Dendritic cells resting, B cells naïve, and Eosinophils (Supplementary Figure S1D). Next, we examined the expression levels of some immune checkpoint molecules in the two clusters and the association of immune checkpoint molecules with prognostic chemokine-related lncRNAs. The expression levels of PD-L1, CTLA4, LAG3, PDCD1LG2, HAVCR2, SIGLEC15, and TIGIT were remarkably higher in cluster 2 than in cluster 1 (Figures 3(e)–3(k)). Furthermore, PD-L1, CTLA4, LAG3, PDCD1LG2, HAVCR2, and TIGIT were positively correlated with multiple prognostic chemokine-related lncRNAs, and only SIGLEC15 was negatively correlated with the expression of some chemokine-related lncRNAs (Figure 3(l)). Thus, we found that two chemokine-related lncRNAs clusters were observably associated with TME and immune checkpoint molecules.

### 3.3. Construction and Validation of the Risk Model for COAD Patients

**Figure 4:**
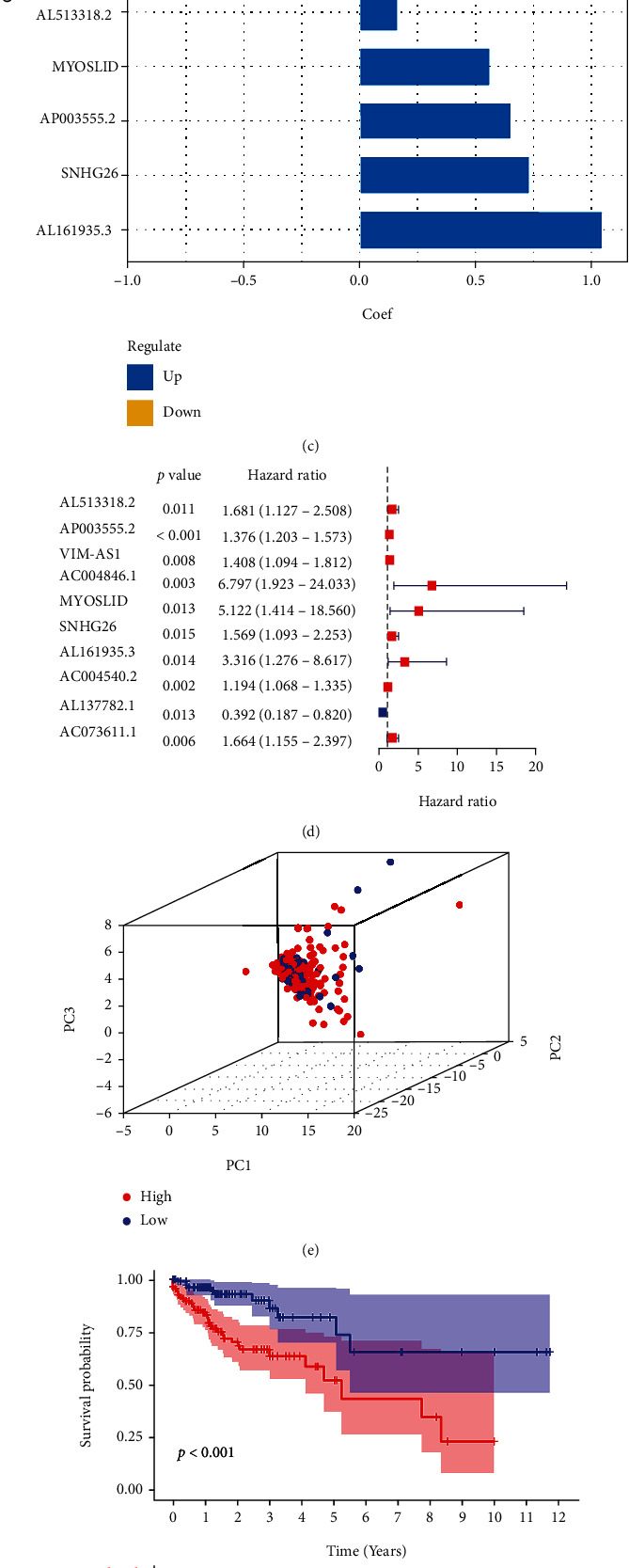
Construction of risk model in COAD. (a and b) The LASSO Cox regression analysis was conducted to construct the risk model. (c) The corresponding coefficients of 10 prognostic chemokine-related lncRNAs in the risk model. (d) Forest plot of 10 prognostic chemokine-related lncRNAs in the risk model. (e) Principal component analysis (PCA) for the entire gene cohort. (f and g) Kaplan–Meier survival curve for the high-risk or low-risk groups in training cohort (f) and test cohort (g). (h and i) ROC curve for predicting 1-, 2-, and 3-year survival rates of the high-risk or low-risk in training cohort (h) and test cohort (i).

To identify the most potent prognostic signature, the lasso regression analyses were performed to identify potential survival-related chemokine-related lncRNAs, resulting in the ten best candidates (Figures 4(a) and 4(b)). The 473 COAD patients were randomized into training and test cohorts, and a risk score was calculated for each patient and then divided into high- and low-risk groups based on the median risk score. The training cohort was used for the establishment of the risk model. The coef value of each lncRNA is shown in Figure 4(c). Ten lncRNAs were identified to construct the prognosis signature in COAD (Figure 4(d)). Based on the risk pattern of the risk model, we performed dimensionality reduction for the whole gene, 64 chemokine-related genes, and genes in the risk model by using principal component analysis (PCA) (Figure 4(e)). Survival analysis showed that the prognosis of COAD patients in the high-risk group was worse than that in the low-risk group in both the training cohort (Figure 4(f)) and the test cohort (Figure 4(g)). To test the accuracy of the risk model in predicting survival, the ROC curve revealed that prognostic chemokine-related lncRNAs accurately predicted overall survival in the training cohort, with AUCs of 0.730, 0.773, and 0.806 for 1-, 3-, and 5-year overall survival rates (Figure 4(h)). ROC results also displayed a curve (AUC) of 0.680, 0.781, and 0.697 for the test cohort's 1-, 3-, and 5-year overall survival rates (Figure 4(i)).

**Figure 5:**
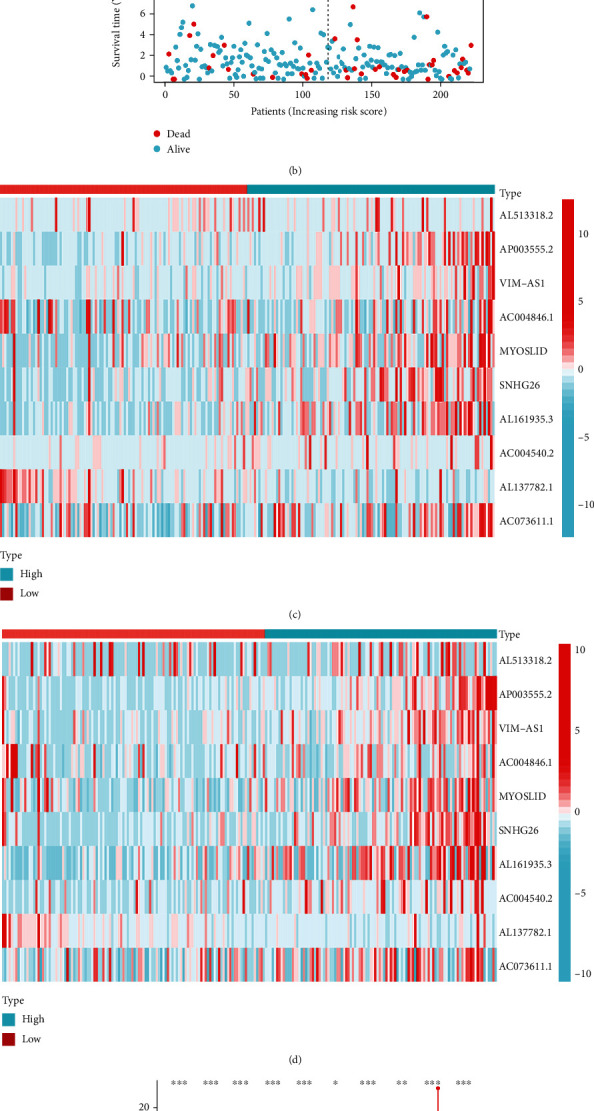
Validation of the risk model in the training and testing groups. (a and b) Distributions of risk score and survival status in the training cohort (a) and the test cohort (b). (c and d) Heatmap of expression levels of chemokine-related prognostic lncRNAs in the training cohort (c) and the test cohort (d). (e) Boxplot of the expression levels of 10 prognostic chemokine-related lncRNAs in tumor and normal tissues. (f) Heatmap of correlation analysis between risk score and clinical parameters. ^∗^*p* < 0.05; ^∗∗^*p* < 0.01; ^∗∗∗^*p* < 0.001.

Subsequently, we achieved risk curves and assessed the survival status and risk of prognostic chemokine-related lncRNAs (Figures 5(a) and 5(b)). As the risk scores increased, the number of deaths and the high-risk patient ratios enhanced. The expression of protective lncRNAs (AC004846.1 and AL137782.1) decreased with increasing risk scores, while the expression of risk lncRNAs (AL513318.2, AP003555.2, VIM-AS1, MYOSLID, SNHG26, AL161935.3, AC004540.2, and AC073611.1) increased with increasing risk scores (Figures 5(c), 5(d)). Thus, our risk model had excellent distinguishing performance in predicting the prognosis and risk of COAD patients. Furthermore, we found ten prognostic chemokine-related lncRNAs were expressed differently in tumor and normal tissues and displayed as a vioplot (Figure 5(e)). The heat map revealed the significant differences in the grade, pT, and clinical stage between the high- and low-risk groups, disclosing a close correlation between clinical features and the risk model. The COAD patients with pT3-4 and G3 had higher risk scores than pT1-2 and G1. Likewise, the risk scores improved obviously as the clinical stage increased from stage I to stage IV (Figure 5(f)). These data suggested that the risk score was dramatically associated with the clinical characteristics of COAD patients. The above findings demonstrated that this risk model has robust and stable predictive power.

### 3.4. Independent Prognostic Factors and Clinicopathological Correlations of the Risk Model

**Figure 6:**
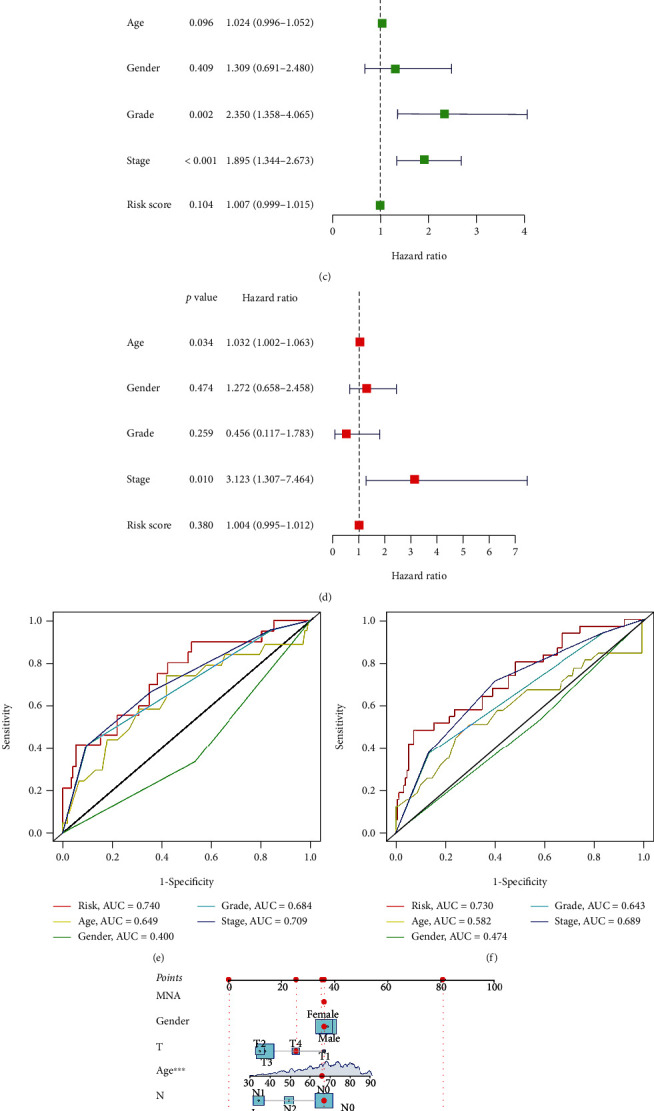
Independent prognosis value of the risk model. (a) Univariate Cox regression analysis of risk score and clinical parameters in the training cohort. (b) The independent prognosis value of the risk score in the training group was validated by multivariate Cox regression analyses. (c and d) Univariate (c) and multivariate (d) Cox regression analysis of risk score and clinical parameters in the test cohort. (e and f) ROC curves showed the superiority of the risk score in predicting the survival rate of patients in the training group (e) and the test group (f). (g) The nomogram for predicting the overall survival of patients based on risk score, risk, age, gender, grade, and stage. (h) Calibration plots of the nomogram for predicting the overall survival probability at 1, 3, and 5 years. (i) ROC curves were plotted to determine the accuracy of the nomogram for overall survival at 1, 3, and 5 years.

We further validated the correlation between the risk model and clinical features of COAD patients. Univariate Cox regression analysis revealed that age, grade, clinical stage, and risk score were associated with the prognosis of COAD patients in the training cohort (Figure 6(a)). Multivariate Cox regression analysis showed that age and risk scores were markedly correlated with the survival outcomes of COAD patients in the training cohort (Figure 6(b)). However, both univariate and multivariate analyses exhibited that the risk score was not associated with the prognosis of COAD patients in the test cohort (Figures 6(c) and 6(d)). To further explore whether the risk model is superior to other clinical features in terms of prognostic predictive role, the ROC curve confirmed that the risk model had higher efficiency than other clinical features in the training and test cohorts (Figures 6(e) and 6(f)). The nomograms and calibration curves were developed to quantify the prediction of individual survival probability at 1-, 3-, and 5 years (Figure 6(g)). The consistency index (C-index) and ROC of the nomogram were acquired to verify the accuracy and validity of the nomogram. We derived a C-index of 0.792 for the nomogram associated with multiple clinical parameters. The calibration curve revealed that the predicted overall survival was largely consistent with the actual observations at 1-, 3-, and 5 years (Figure 6(h)). For the ROC of the overall survival nomogram, the AUC values were 0.680, 0.737, and 0.697 at 1-, 3-, and 5 years, respectively (Figure 6(i)). Subsequently, the stratified survival analysis was applied to evaluate the predictive ability of the risk model for patients with different clinical parameters. Interestingly, we observed that patients in the low-risk group had better survival outcomes than those in the high-risk group in all subgroups. Details were as follows: among COAD patients with aged >60, aged ≤60 years old, female, male, tumor grade 1-2, tumor grade 3, pT3-4, pN0, pN1-2, pM0, pM1, stage I-II, and stage III-IV, the high-risk group had a worse prognosis than the low-risk group (Supplementary Figure S2). These clinical data analyses confirmed the good predictive performance of the risk model.

### 3.5. Analysis of Pathways Associated with the Risk Model

**Figure 7:**
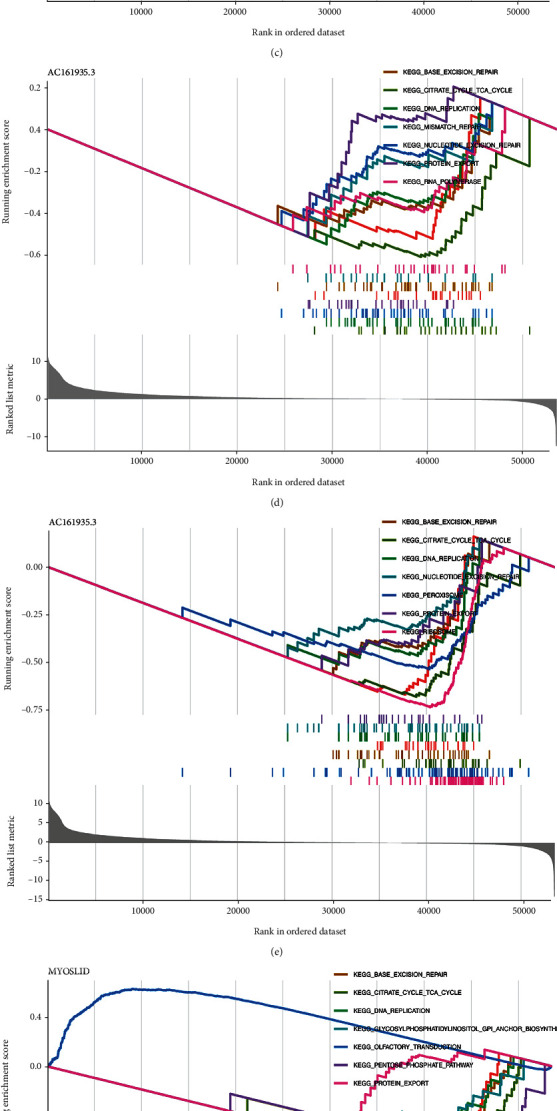
Correlation between pathways and risk model. (a) GSVA results based on the chemokine-related lncRNAs risk model. (b) GSEA results based on the chemokine-related lncRNAs risk model. ^∗^*p* < 0.05, ^∗∗^*p* < 0.01, ^∗∗∗^*p* < 0.001.

In this model, multiple active pathways were gained in high-risk or low-risk groups to study the KEGG pathway enriched by risk scores and model lncRNAs. GSVA results revealed numerous carcinogenic- and immune-related signaling pathways were noteworthily associated with chemokine-related lncRNAs and risk scores. For example, there was a positive correlation between the VEGF, Toll-like receptor, TGF-*β*, T cell receptor, Nod-like receptor, MAPK, JAK-STAT, and B cell receptor and patient risk scores, and these pathways were active in the high-risk group (Figure 7(a)). Then, gene set enrichment analysis (GSEA) was performed to ascertain the enrichment pathways in low-risk patients. These pathways included the base excision repair, DNA replication, citrate cycle, pentose phosphate pathway, protein export, nonhomologous end joining, selenium amino acid metabolism, ribosome, steroid biosynthesis, RNA polymerase, mismatch repair, and endometrial cancer (Figures 7(b)–7(h)). We found that this risk model was associated with tumor- and immune-related pathways in COAD.

### 3.6. Correlation Analysis of the Risk Model with Immune Cell Infiltration and TME

**Figure 8:**
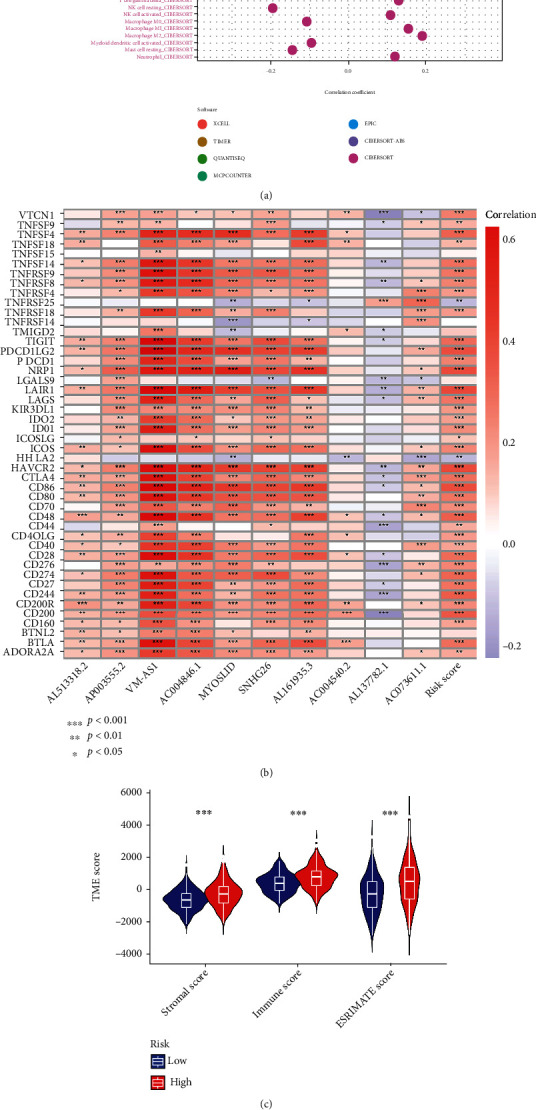
Correlation analysis of the risk model with immune cell infiltration and TME. (a) Bubble plot of the correlation between the risk model and tumor immune cells in COAD patients. (b) Heatmap of the associations between expression levels of multiple immune checkpoints and risk score. (c) Correlation of risk model and ESTIMATEScore, immuneScore, and stromalScore. (d) The difference in the enrichment of 13 immune-related pathways between the low-risk and high-risk groups. ^∗^*p* < 0.05, ^∗∗^*p* < 0.01, ^∗∗∗^*p* < 0.001.

Here, we used various software to calculate the infiltration status of COAD samples and obtain immune cell infiltration values. First, the correlation analysis of immune cell infiltration and risk score was calculated, showing that multiple immune cell infiltrations were associated with patient risk scores (Supplementary Table S12). The correlation bubbles displayed that the following immune cell infiltration levels were positively correlated with the risk score: memory B cells, naive B cells, naive CD4+ T cells, CD8+ T cells, monocyte, macrophage M1, myeloid dendritic cells activated, and activated mast cells. However, infiltration levels of resting NK cells and resting mast cells were inversely associated with the risk score (Figure 8(a)). We then examined significant positive associations between expression levels of multiple immune checkpoints and risk scores (Figure 8(b)). In the TME, the average immuneScore, stromalScore, and ESTIMATEScore were markedly higher in the high-risk group than in the low-risk group (Figure 8(c)). Thus, the immune-related data of the risk model were partially consistent with the chemokine-related lncRNAs cluster analysis. Next, we identified differences in 13 immune-related pathways between the high- and low-risk groups. The ssGSEA analysis indicated that 12 of the 13 pathways dramatically differed between the high-risk and low-risk groups, and these 12 pathways were more active in the high-risk group (Figure 8(d)). In addition, we examined numerous model molecules associated with immune cell infiltration, including VIM-AS1, AC004846.1, MYOSLID, and AL161935.3 (Supplementary Table S13). Our findings suggested that the risk model was closely correlated to immune cell infiltration and TME, which could predict immune cell infiltration and TME in COAD to a certain extent.

### 3.7. Correlation between Risk Model and Tumor Mutational Burden

**Figure 9:**
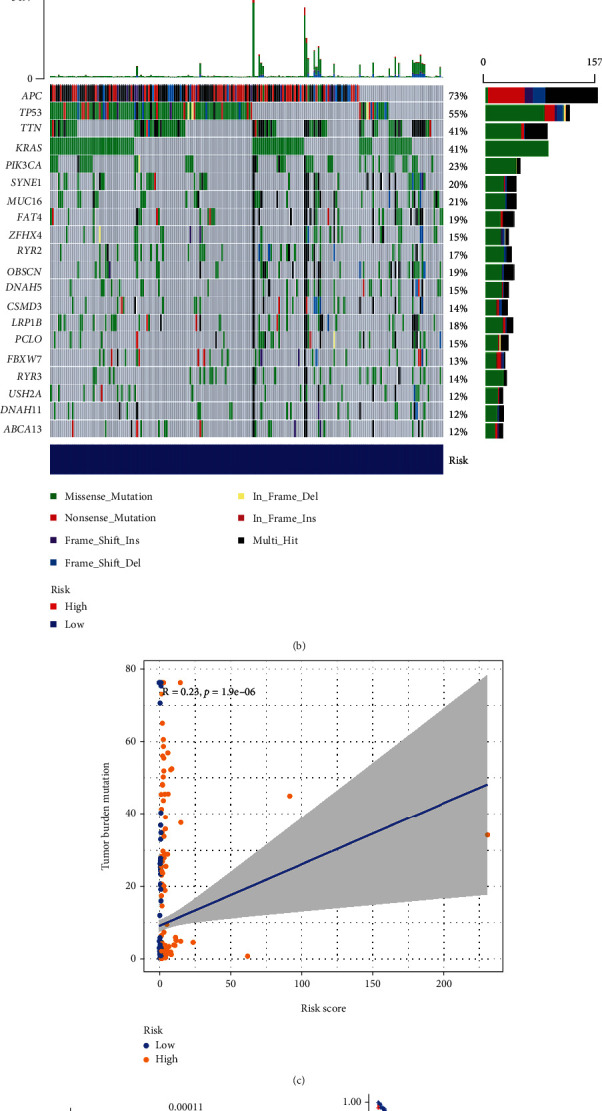
Correlation between the risk model and TMB. (a and b) Waterfall chart of the top 20 genes with increased mutation frequency in high- and low-risk groups. (c) Scatter plot of positive correlation between risk score and TMB. (d) Boxplot of the expression level of TMB between the low-risk and high-risk groups. (e) The survival curve of the difference between the high TMB group and the low TMB group. (f) The survival status of patients with low-risk or high-risk scores in the high TMB and low TMB groups.

We downloaded TMB data from TCGA-COAD samples using R's “maftools” and divided the TMB data into high-risk and low-risk data based on the risk score. The TMB status was then calculated and analyzed in the high-risk and low-risk groups. Except for APC, TP53, and LRP1B molecules, the mutation rate in the high-risk group was more than 5% higher than that in the low-risk group (Figures 9(a) and 9(b)). We observed the risk score was positively associated with TMB levels (Figure 9(c)). We also compared the differences in TMB between low-risk and high-risk groups, and the results revealed that patients in the high-risk group had higher TMB levels than in the low-risk group (Figure 9(d)). We divided the patients into the high-TMB and low-TMB groups based on the TMB levels and analyzed survival outcomes. The results indicated that patients with high TMB had a poor prognosis compared with patients with low TMB (Figure 9(e)). COAD patients with high-risk scores in the high TMB group had the worst survival outcomes. COAD patients in the low TMB group with high-risk scores also had worse survival outcomes than low TMB with low-risk scores (Figure 9(f)). Thus, the risk model was associated with TMB and prognosis.

### 3.8. Analysis of Drug Sensitivity and Immunotherapy in the Risk Model

**Figure 10:**
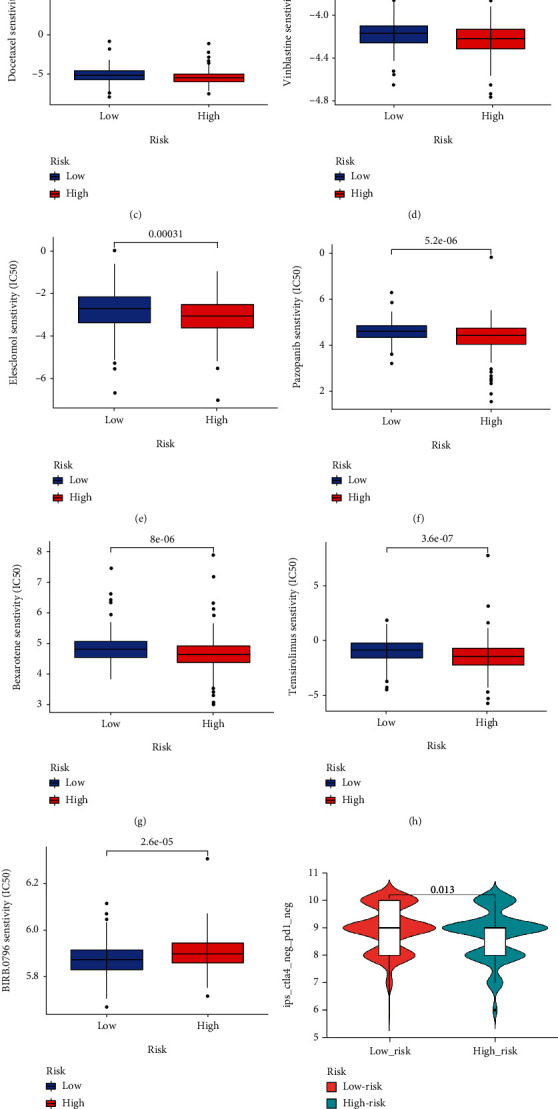
Drug sensitivity and immunotherapy in the risk model. (a–i) Risk stratification showed the responses of high- and low-risk groups to multiple drugs. (j and k) The immunotherapy scores of patients with a single positive for CTLA4 and negative for both PD-L1 and CTLA4 in the low-risk patients were higher than that of high-risk patients. ^∗^*p* < 0.05, ^∗∗^*p* < 0.01, ^∗∗∗^*p* < 0.001.

Chemotherapy and targeted therapy are current strategies to treat COAD; it is critical to understand the effectiveness and sensitivity of these drugs to different risk groups. We predicted the sensitivities to common anticancer drugs, chemotherapeutics, and targeted agents in high- and low-risk groups of COAD patients. The IC50 values of Camptothecin, Cisplatin, Docetaxel, Vinblastine, Elesclomol, Pazopanib, Bexarotene, and Temsirolimus in the high-risk group were lower than those in the low-risk group, indicating that these drugs are more sensitive to the high-risk patients (Figures 10(a)–10(h)). In contrast, the low-risk group was more sensitive to BIRB.0796 (Doramapimod) (Figure 10(i)). Risk stratification also revealed remarkable differences in drug sensitivity between high- and low-risk groups for many other drugs (Supplementary Figure S3). Next, we downloaded immunotherapy score data from the TCIA database and obtained the difference in immunotherapy scores between high- and low-risk groups. The results exhibited that low-risk patients who were single positive for CTLA4 and negative for both PD-L1 and CTLA4 had higher immunotherapy scores, indicating that the patients in the low-risk group would benefit from immunotherapy (Figures 10(j)–10(k)). Therefore, our risk model was a potential target for multiple drugs and had vital implications for guiding the personalized treatment of patients with COAD.

### 3.9. Prognostic Value and Expression of each lncRNA in our Risk Model

**Figure 11:**
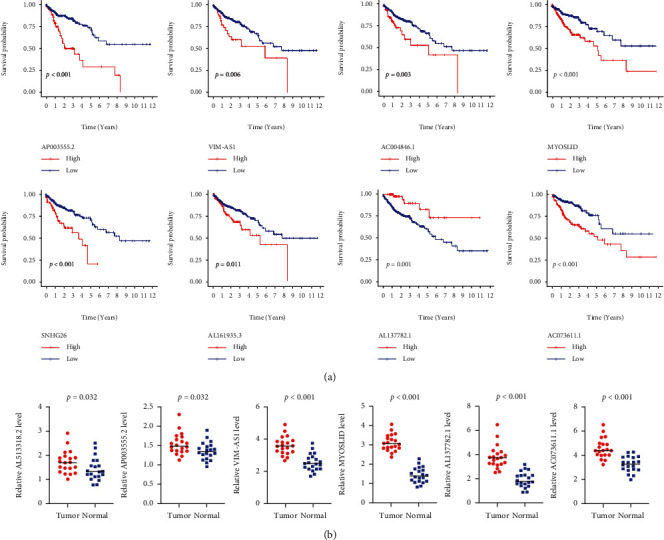
Prognostic value and expression of each lncRNA in our risk model. (a) The risk model's prognostic value of each lncRNA in COAD patients. (b) Expression levels of each lncRNA between tumor and adjacent normal tissues in 20 clinic samples. ^∗^*p* < 0.05, ^∗∗^*p* < 0.01, ^∗∗∗^*p* < 0.001.

To validate more valuable lncRNAs in the risk model, we further examined each lncRNA's prognostic value and expression in COAD patients. Except for AL137782.1, the remaining high-expressing lncRNAs had a worse prognosis in COAD patients than low-expressing lncRNAs, indicating the expression levels of most lncRNAs in the risk model guide patient prognosis (Figure 11(a)). Next, we collected 20 COAD tumors and adjacent normal samples and then conducted the qRT-PCR assays to examine the expression levels of these lncRNAs in clinical samples. Six of ten lncRNAs were differentially expressed between tumor and normal samples, including AL513318.2, AP003555.2, VIM-AS1, MYOSLID, AL137782.1, and AC073611.1 (Figure 11(b)). The expression trends were consistent with those listed in TCGA-COAD data. These results suggested that the most lncRNAs of this risk model might exert a more vital function in COAD.

## 4. Discussion

Numerous studies have reported that chemokine modification events are involved in tumor progression, including promoting cancer cell differentiation or regulating tumor formation and metastasis potential [10, 23]. Studies have also emphasized that chemokines regulate multiple biological processes, including mammalian development, stem cell renewal, immune responses, drug resistance, and tumor progression [24]. For example, Zeng et al. discovered that the CCL5/CCR5 axis is involved in the pathological processes of different diseases such as inflammation, chronic diseases, cancer, and infection of COVID-19 and the related signaling pathways of its regulatory axis [25]. Chen et al. found that the CXCL2/CXCR2 axis induced cancer stem cell signatures in CPT-11-resistant LoVo colon cancer cells [26]. Due to the limited predictive power of general prognostic models, a novel prognostic chemokine-related lncRNAs model could improve the monitoring and management of malignancies such as COAD. The study of prognostic chemokine-related lncRNAs is of great significance for guiding the direction and goals of COAD research. In this study, we first explored the differences between the survival outcomes and immune status of two chemokine-related lncRNAs clusters by consensus clustering. We then constructed a risk model of prognostic chemokine-related lncRNAs and validated the validity and accuracy of the model in predicting survival and clinical parameters in COAD patients. Our further analysis showed that the risk model played a vital role in predicting immune cell infiltration, immune checkpoint gene expression, tumor mutational burden, immunotherapy score, and drug sensitivity in COAD patients. Furthermore, we analyzed the prognostic value and expression of each lncRNA in this model in COAD patients. This study provided clues for COAD progression and treatment by comprehensively analyzing the characteristics of novel prognostic chemokine-related lncRNAs associated with the immune landscape.

We first obtained RNA-seq profiles of 473 COAD samples from the TCGA dataset and extracted the chemokine-related lncRNAs data. Fifty-two prognostic chemokine-related lncRNAs were identified in COAD patients by univariate Cox regression analysis. By consensus clustering, COAD patients were classified into subgroups based on the consistent expression of prognostic chemokine-related lncRNAs. COAD patients in cluster 2 had worse overall survival than patients in cluster 1, suggesting that the prognostic chemokine-related lncRNAs cluster affects the survival of COAD patients. Clinical correlation analysis revealed no significant differences in the clinical parameters of COAD patients between the two clusters. Afterward, we demonstrated that COAD patients in cluster 2 had higher immune scores, stromal scores, and ESTIMATE scores than those in cluster 1, suggesting a higher degree of immune infiltration in cluster 2 than in cluster 1. These findings were similar to previous studies that demonstrated lower overall survival in patients with tumors with high immune and stromal scores [27, 28]. We also found that multiple immune checkpoint molecules were expressed at higher levels in cluster 2 than in cluster 1, implying that the clustering pattern of chemokine-related lncRNAs is closely related to TME. Jin et al. reported that various lncRNAs could indirectly regulate the expression of immune checkpoint molecules, thereby affecting the survival outcomes of tumor patients [29]. Thus, we speculated that cluster 2 might enhance the expression of immune checkpoint molecules through numerous pathways, causing the decreased overall survival of COAD patients.

To further explore the role and value of chemokine-related lncRNAs in COAD, the 10 chemokine-related lncRNAs were identified to construct the risk model using LASSO Cox regression analysis. Survival analysis found that in the training set, the survival outcomes of patients in the high-risk group were worse than those in the low-risk group. The AUC value of the ROC curve confirmed the risk model's efficiency and accuracy for the training and the test cohorts. The high-risk and the low-risk groups also showed significant differences in grades, pT, and clinical stage, and the risk model also showed close correlations between clinical parameters. Then, the expression levels of 10 prognostic chemokine-related lncRNAs differed between tumor and normal tissues. Tu et al. showed that TCF4 enhanced colorectal cancer liver metastasis by regulating tumor-associated macrophages through the CCL2/CCR2 signaling pathway [30]. Jie et al. reported that targeting KDM4C enhanced CD8 T cell-mediated antitumor immunity by activating the transcription of the chemokine CXCL10 in lung cancer [31]. The above findings nicely explained that some chemokine-related lncRNAs are overexpressed in tumors to act as oncogenes, while others are highly expressed in normal tissues as tumor suppressors. Subsequently, we explored that the risk score of the risk model was an independent prognostic factor in predicting the survival outcomes of COAD patients. ROC curves were also performed to validate risk score accuracy in independent prognostic functions. All patients with different clinical characteristics in the high-risk group had worse survival outcomes than in the low-risk group. Next, the nomogram predicted survival time was almost consistent with the actual survival time. For example, Liang et al. reported that the chemokine signature was identified for predicting overall survival in gastric cancer and showed good predictive efficiency, similar to our model [32]. In total, our risk model has sufficient efficiency and accuracy in predicting the survival outcomes of COAD patients.

Recent studies have reported that chemokines modifications and multiple lncRNAs can modulate the process of cancer immunity, including immune cell infiltration and immune resistance and activation in the TME, causing tumor progression [33, 34]. Thus, to explore whether the risk model played a role in tumor and TME, we first performed GSEA and GSVA analyses. Multiple cancer- and immune-related pathways were associated with the risk model, such as the VEGF, Toll-like receptor, TGF-*β*, T cell receptor, Nod-like receptor, MAPK, JAK-STAT, and B cell receptor, base excision repair, DNA replication, citrate cycle, pentose phosphate pathway, protein export, nonhomologous end joining, selenium amino acid metabolism, ribosome, steroid biosynthesis, RNA polymerase, mismatch repair, endometrial cancer, and these pathways were enriched in the high-risk group. Also, some studies have proved that these pathways could regulate immune cell infiltration and TME [23, 35]. Based on this, we considered that the risk model was likely to affect cancer immune processes in COAD, including immune cell infiltration, immune resistance and activation, and immune checkpoint molecules. Then, we found that the infiltration levels of memory B cells, naive B cells, naive CD4+ T cells, CD8+ T cells, monocyte, macrophage M1, myeloid dendritic cells activated, and activated mast cells were positively associated with the risk score, suggesting that these cells were more infiltrated in high-risk patients. Other resting NK cells and resting mast cells were increased in the low-risk patients, meaning that low-risk patients have more infiltration of these cells. Studies have reported that tumor patients have a variety of immune cell infiltration involved in tumor progression. For example, patients with more CD4+ and CD8+ T cell infiltration responded better and benefited from immunotherapy [36]. The massive infiltration of macrophages in solid tumors can promote tumor progression and distant metastasis, resulting in poor patient survival and weak treatment outcomes [37]. Furthermore, the high-risk patients had higher immune, stroma, and ESTIMATE scores than the low-risk patients, indicating that the TME in the high-risk group had more immune infiltration than the low-risk group. These data were similar to previous studies showing that malignancies with high immune and stromal scores had a worse overall survival [38]. The literature also suggested that more tumor-infiltrating immune cells in the high-risk group were associated with an increased risk of recurrence and poorer survival [39]. Thus, we speculated that lower immunoreactivity and higher immunosuppression in the TME would cause worse survival in high-risk patients. These results supported this risk model as a predictor of immune landscape in COAD patients.

The expression levels of immune checkpoint molecules and TMB are considered effective immunotherapy indicators. Studies have shown that gastric cancer patients with higher immune checkpoint gene expression and higher somatic mutations have better effects on immunotherapy [40]. We determined the expression of immune checkpoint molecules corresponding to risk score and model lncRNAs; the data revealed that the most immune checkpoint molecules were significantly associated with high-risk patients, suggesting that risk score was closely related to immune status. Therefore, we speculate that high-risk patients may be more sensitive to immunotherapy. Risk scores were subsequently found to be positively correlated with TMB, suggesting that patients with high-risk scores had higher levels of TMB. Meanwhile, high-risk patients with high TMB also displayed the worst prognostic outcomes. Kim et al. constructed a novel TME signature and found that gastric cancer progression may be affected by TME and frameshift mutations, similar to our model [41]. Thus, this risk model was strongly associated with immune cell infiltration and TMB. However, further study is required to investigate whether immune cell infiltration is affected by TMB.

Jung et al. reported that the clinical utility of chemotherapy and targeted agents for COAD had been extensively studied [42]. Afterward, we assessed the sensitivity and efficacy of immunotherapy in high- and low-risk groups. The low-risk patients who were single positive for CTLA4 and negative for both PD-L1 and CTLA4 had higher immunotherapy scores, meaning that these low-risk patients would benefit from immunotherapy. The high-risk group was also markedly associated with susceptibility to multiple targeted drugs, including commonly used clinical chemotherapy cisplatin, docetaxel, vinblastine, and some novel drugs. These data suggested that this prognostic chemokine-related lncRNAs risk model has potential utility in estimating efficacy and sensitivity to various medications. To validate a reliable and accurate risk model, we not only investigated the correlation between the expression of each lncRNA and patient prognosis but also detected the expression levels of lncRNAs in clinical samples. Many highly expressed lncRNAs have a worse prognosis in COAD patients than lowly expressed lncRNAs. Many lncRNAs in the risk model were expressed differently between tumor and normal tissues, and these expression trends were consistent with the trend in the TCGA-COAD data. These findings further demonstrated that lncRNAs in the risk model have more excellent research value.

Nonetheless, the current study has some limitations. The risk model was created using public data and lacks enough clinical samples and data. The expression of 10 chemokine-related lncRNAs containing this signature was validated on only 20 pairs of clinical samples. Furthermore, the biological functions and mechanisms of prognostic chemokine-related lncRNAs in COAD remained uncertain, and experimental studies were needed to verify these findings. Based on this, we will expand the sample size for validation and conduct further experimental studies.

## 5. Conclusions

In conclusion, we first explored the differences between the survival outcomes and immune status of two chemokine-related lncRNAs clusters by consensus clustering. We then constructed a novel risk model of prognostic chemokine-related lncRNAs and validated the validity and accuracy of the model in predicting survival and clinical parameters in COAD patients. The risk model also played a vital role in predicting immune cell infiltration, TME, TMB, immunotherapy, and drug sensitivity in COAD patients. These findings elucidated the critical role of novel prognostic chemokine-related lncRNAs in prognosis, immune landscape, and drug therapy, thereby providing valuable insights for prognosis assessment and personalized treatment strategies for COAD patients.

## Data Availability

The datasets supporting the conclusions of this article are available upon reasonable request from the TCGA database (https://portal.gdc.cancer.gov/), the TCIA database (https://tcia.at/), and the NCI-60 database (https://discover.nci.nih.gov/cellminer/home.do).

## Supplementary Materials

Supplementary Materials Supplementary Figure S1: (A) Heatmap of the expression of prognostic chemokine-related lncRNAs between tumor tissues and normal tissues. (B) Boxplot of the expression of prognostic chemokine-related prognostic lncRNAs in COAD tissues and normal tissues. (C) Barplot showed the proportion of 22 kinds of TIICs in each COAD sample. (D) Correlation between 22 kinds of TIICs was visualized. ^∗^*p* < 0.05; ^∗∗^*p* < 0.01; ^∗∗∗^*p* < 0.001. Supplementary Figure S2: Survival analysis was conducted in high-risk and low-risk patients with different clinical parameters. Supplementary Figure S3: Risk stratification showed the differences in drug susceptibility between high-risk and low-risk groups. Supplementary Table S1: Clinical information of the COAD patients. Supplementary Table S2: The 64 chemokine genes. Supplementary Table S3: Expression of 52 chemokine-related lncRNAs in COAD. Supplementary Table S4: Immune cell content of COAD samples was calculated using the CIBERSORT algorithm. Supplementary Table S5: Immune, Stromal, and ESTIMATE scores for COAD samples. Supplementary Table S6: Expression of 10 chemokine-related lncRNAs and risk score in the entire cohort. Supplementary Table S7: The immune cell infiltration status in all tumor samples in RNA-seq. Supplementary Table S8: Tumor mutation burden data of the low-risk group. Supplementary Table S9: Tumor mutation burden data of the high-risk group. Supplementary Table S10. The immunotherapy score data was obtained from the TCIA database. Supplementary Table S11: Quantitative real-time PCR primer sequences of model lncRNAs. Supplementary Table S12: The correlation values of immune cell infiltration and patient risk score. Supplementary Table S13: The correlation values of chemokine-related lncRNAs and immune cells.
