# Supplementary material for: A Novel Prognostic Chemokine-Related lncRNAs Signature Associated with Immune Landscape in Colon Adenocarcinoma
Source: Dis Markers. 2022 Nov 3;2022:2823042. doi: 10.1155/2022/2823042 (PMC9649319; doi:10.1155/2022/2823042)
Supplement: Supplementary Materials — Supplementary Figure S1: (A) Heatmap of the expression of prognostic chemokine-related lncRNAs between tumor tissues and normal tissues. (B) Boxplot of the expression of prognostic chemokine-related prognostic lncRNAs in COAD tissues and normal tissues. (C) Barplot showed the proportion of 22 kinds of TIICs in each COAD sample. (D) Correlation between 22 kinds of TIICs was visualized. ∗p < 0.05; ∗∗p < 0.01; ∗∗∗p < 0.001. Supplementary Figure S2: Survival analysis was conducted in high-risk and low-risk patients with different clinical parameters. Supplementary Figure S3: Risk stratification showed the differences in drug susceptibility between high-risk and low-risk groups. Supplementary Table S1: Clinical information of the COAD patients. Supplementary Table S2: The 64 chemokine genes. Supplementary Table S3: Expression of 52 chemokine-related lncRNAs in COAD. Supplementary Table S4: Immune cell content of COAD samples was calculated using the CIBERSORT algorithm. Supplementary Table S5: Immune, Stromal, and ESTIMATE scores for COAD samples. Supplementary Table S6: Expression of 10 chemokine-related lncRNAs and risk score in the entire cohort. Supplementary Table S7: The immune cell infiltration status in all tumor samples in RNA-seq. Supplementary Table S8: Tumor mutation burden data of the low-risk group. Supplementary Table S9: Tumor mutation burden data of the high-risk group. Supplementary Table S10. The immunotherapy score data was obtained from the TCIA database. Supplementary Table S11: Quantitative real-time PCR primer sequences of model lncRNAs. Supplementary Table S12: The correlation values of immune cell infiltration and patient risk score. Supplementary Table S13: The correlation values of chemokine-related lncRNAs and immune cells. [file 2823042.f1.zip › Supplementary Figure 2 (1).pdf]

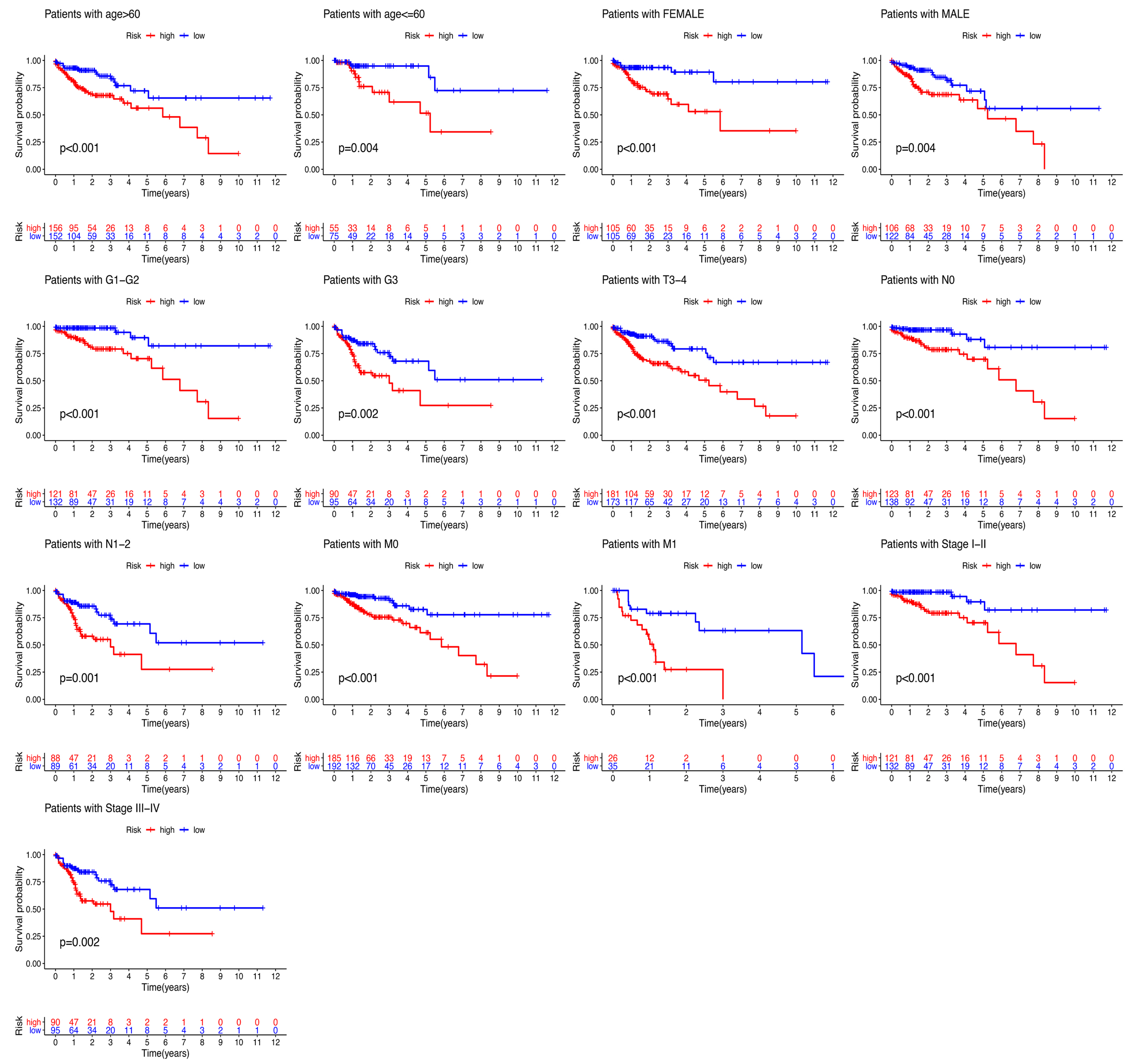

Patients with age>60
Risk high low
Survival probability
p<0.001
Time(years)
Risk high 156 95 54 26 13 8 6 4 3 1 0 0 0
low 152 104 59 33 16 11 8 8 4 4 3 2 0
Patients with age<=60
p=0.004
Risk high 55 33 14 8 6 5 1 1 1 0 0 0 0
low 75 49 22 18 14 9 5 3 3 2 1 1 0
Patients with FEMALE
p<0.001
Risk high 105 60 35 15 9 6 2 2 2 1 0 0 0
low 105 69 36 23 16 11 8 6 5 4 3 2 0
Patients with MALE
p=0.004
Risk high 106 68 33 19 10 7 5 3 2 0 0 0 0
low 122 84 45 28 14 9 5 5 2 2 1 1 0
Patients with G1−G2
p<0.001
Risk high 121 81 47 26 16 11 5 4 3 1 0 0 0
low 132 89 47 31 19 12 8 7 4 4 3 2 0
Patients with G3
p=0.002
Risk high 90 47 21 8 3 2 2 1 1 0 0 0 0
low 95 64 34 20 11 8 5 4 3 2 1 1 0
Patients with T3−4
p<0.001
Risk high 181 104 59 30 17 12 7 5 4 1 0 0 0
low 173 117 65 42 27 20 13 11 7 6 4 3 0
Patients with N0
p<0.001
Risk high 123 81 47 26 16 11 5 4 3 1 0 0 0
low 138 92 47 31 19 12 8 7 4 4 3 2 0
Patients with N1−2
p=0.001
Risk high 88 47 21 8 3 2 2 1 1 0 0 0 0
low 89 61 34 20 11 8 5 4 3 2 1 1 0
Patients with M0
p<0.001
Risk high 185 116 66 33 19 13 7 5 4 1 0 0 0
low 192 132 70 45 26 17 12 11 7 6 4 3 0
Patients with M1
p<0.001
Risk high 26 12 2 1 0 0 0
low 35 21 11 6 4 3 1
Patients with Stage I−II
p<0.001
Risk high 121 81 47 26 16 11 5 4 3 1 0 0 0
low 132 89 47 31 19 12 8 7 4 4 3 2 0
Patients with Stage III−IV
p=0.002
Risk high 90 47 21 8 3 2 2 1 1 0 0 0 0
low 95 64 34 20 11 8 5 4 3 2 1 1 0
