# Supplementary figures and images for: A Novel Prognostic Chemokine-Related lncRNAs Signature Associated with Immune Landscape in Colon Adenocarcinoma
Source: Dis Markers. 2022 Nov 3;2022:2823042. doi: 10.1155/2022/2823042 (PMC9649319; doi:10.1155/2022/2823042)

A

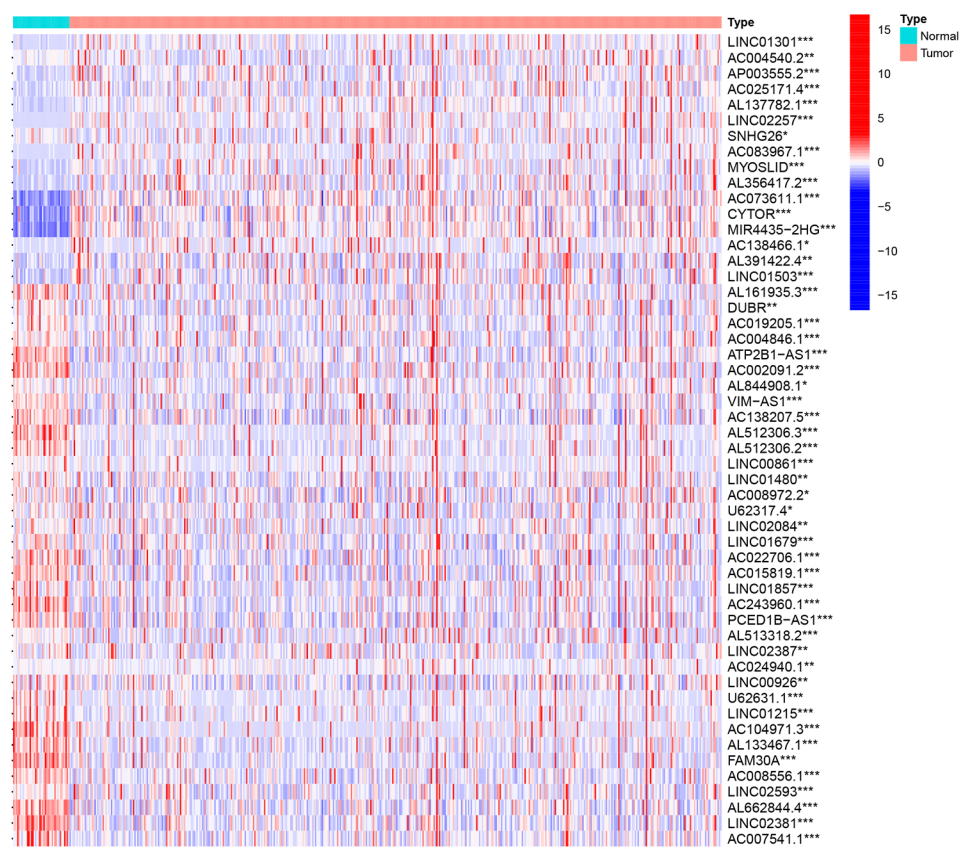

B

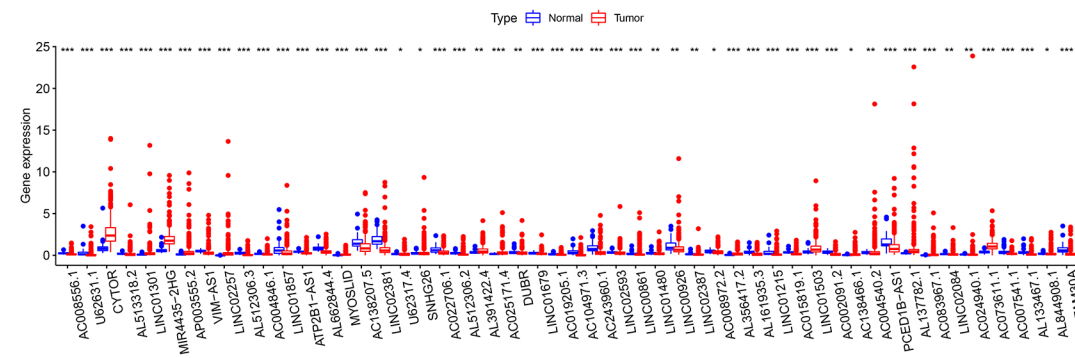

D

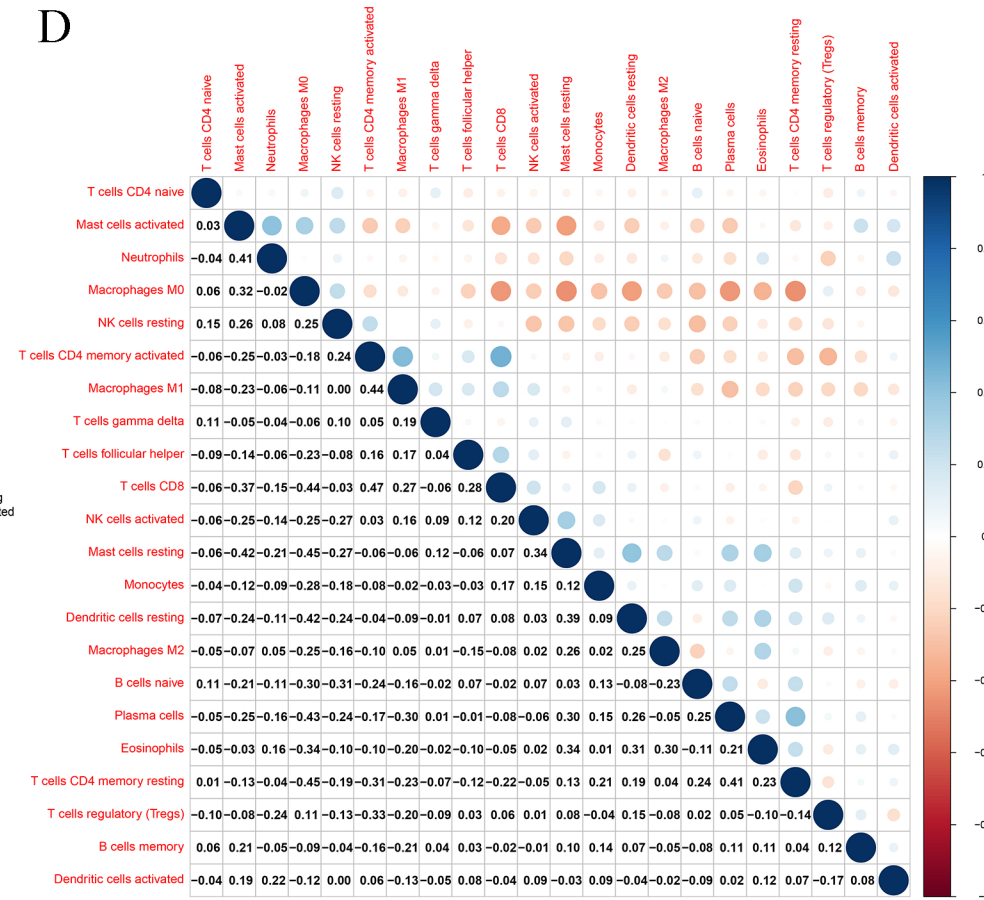

C

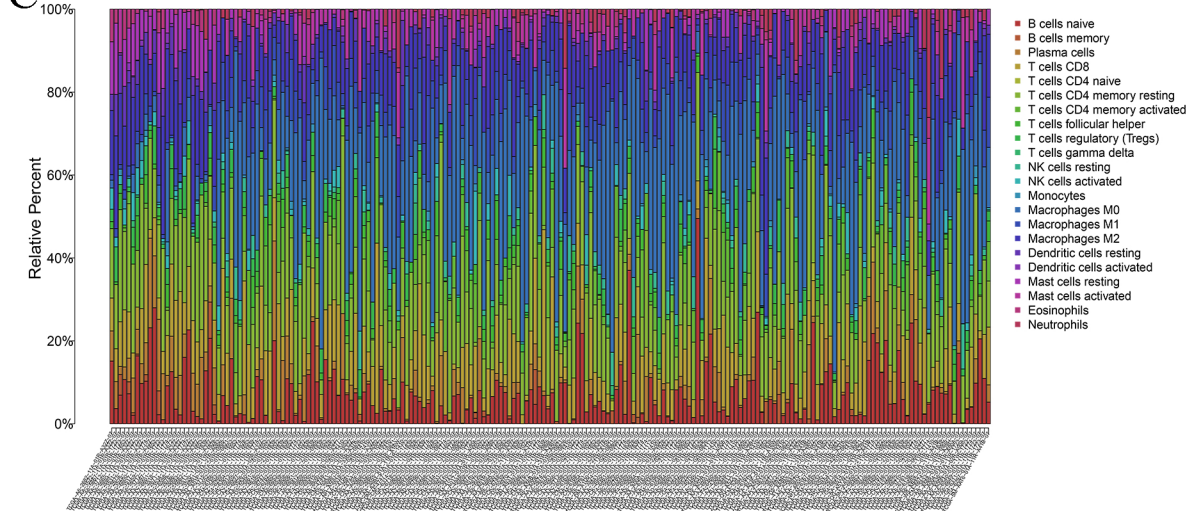

Supplement: Supplementary Materials — Supplementary Figure S1: (A) Heatmap of the expression of prognostic chemokine-related lncRNAs between tumor tissues and normal tissues. (B) Boxplot of the expression of prognostic chemokine-related prognostic lncRNAs in COAD tissues and normal tissues. (C) Barplot showed the proportion of 22 kinds of TIICs in each COAD sample. (D) Correlation between 22 kinds of TIICs was visualized. ∗p < 0.05; ∗∗p < 0.01; ∗∗∗p < 0.001. Supplementary Figure S2: Survival analysis was conducted in high-risk and low-risk patients with different clinical parameters. Supplementary Figure S3: Risk stratification showed the differences in drug susceptibility between high-risk and low-risk groups. Supplementary Table S1: Clinical information of the COAD patients. Supplementary Table S2: The 64 chemokine genes. Supplementary Table S3: Expression of 52 chemokine-related lncRNAs in COAD. Supplementary Table S4: Immune cell content of COAD samples was calculated using the CIBERSORT algorithm. Supplementary Table S5: Immune, Stromal, and ESTIMATE scores for COAD samples. Supplementary Table S6: Expression of 10 chemokine-related lncRNAs and risk score in the entire cohort. Supplementary Table S7: The immune cell infiltration status in all tumor samples in RNA-seq. Supplementary Table S8: Tumor mutation burden data of the low-risk group. Supplementary Table S9: Tumor mutation burden data of the high-risk group. Supplementary Table S10. The immunotherapy score data was obtained from the TCIA database. Supplementary Table S11: Quantitative real-time PCR primer sequences of model lncRNAs. Supplementary Table S12: The correlation values of immune cell infiltration and patient risk score. Supplementary Table S13: The correlation values of chemokine-related lncRNAs and immune cells. [file 2823042.f1.zip › Supplementary Figure 1 (1).pdf]

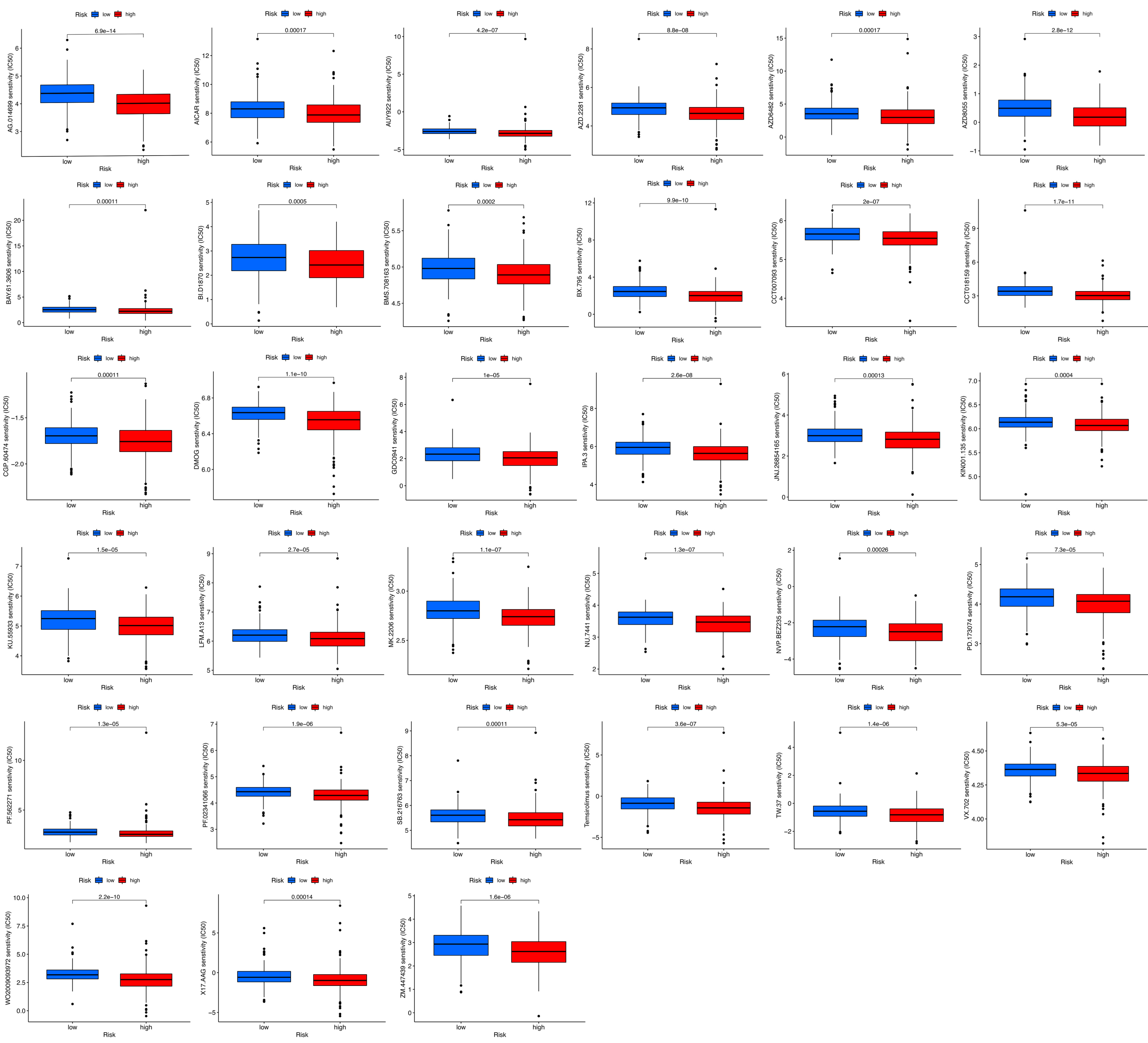

Supplement: Supplementary Materials — Supplementary Figure S1: (A) Heatmap of the expression of prognostic chemokine-related lncRNAs between tumor tissues and normal tissues. (B) Boxplot of the expression of prognostic chemokine-related prognostic lncRNAs in COAD tissues and normal tissues. (C) Barplot showed the proportion of 22 kinds of TIICs in each COAD sample. (D) Correlation between 22 kinds of TIICs was visualized. ∗p < 0.05; ∗∗p < 0.01; ∗∗∗p < 0.001. Supplementary Figure S2: Survival analysis was conducted in high-risk and low-risk patients with different clinical parameters. Supplementary Figure S3: Risk stratification showed the differences in drug susceptibility between high-risk and low-risk groups. Supplementary Table S1: Clinical information of the COAD patients. Supplementary Table S2: The 64 chemokine genes. Supplementary Table S3: Expression of 52 chemokine-related lncRNAs in COAD. Supplementary Table S4: Immune cell content of COAD samples was calculated using the CIBERSORT algorithm. Supplementary Table S5: Immune, Stromal, and ESTIMATE scores for COAD samples. Supplementary Table S6: Expression of 10 chemokine-related lncRNAs and risk score in the entire cohort. Supplementary Table S7: The immune cell infiltration status in all tumor samples in RNA-seq. Supplementary Table S8: Tumor mutation burden data of the low-risk group. Supplementary Table S9: Tumor mutation burden data of the high-risk group. Supplementary Table S10. The immunotherapy score data was obtained from the TCIA database. Supplementary Table S11: Quantitative real-time PCR primer sequences of model lncRNAs. Supplementary Table S12: The correlation values of immune cell infiltration and patient risk score. Supplementary Table S13: The correlation values of chemokine-related lncRNAs and immune cells. [file 2823042.f1.zip › Supplementary Figure 3.pdf]
